# Optimization of virtual water flows in agriculture by changing cropping patterns using an integrated approach

**DOI:** 10.1016/j.heliyon.2023.e22603

**Published:** 2023-11-21

**Authors:** Mukesh Kumar Mehla, Mahesh Kothari, P.K. Singh, S.R. Bhakar, K.K. Yadav

**Affiliations:** a Department of Soil and Water Engineering, College of Technology and Engineering, Maharana Pratap University of Agriculture and Technology, Udaipur, 313001, Raj, India; b Department of Soil Science, Rajasthan College of Agriculture, Maharana Pratap University of Agriculture and Technology, Udaipur, 313001, Raj, India

**Keywords:** Water footprint, Optimizing cropping patterns, Cropping patterns, Sustainable agriculture and water scarcity

## Abstract

Utilizing available water resources efficiently is crucial to address both our present and future requirements and plays a vital role in safeguarding food security. This current investigation deals with assessment and optimizing water footprint (WF) and virtual water flow (VWF) for primary crops in Banas River Basin (BRB) using AquaCrop model with local datasets and district-level estimates. VWF in the basins were estimated by multiplying the WF of crops with the amount exported/imported, which is determined based on the difference between production and consumption in the basin. The possibility of changing the cropping patterns was evaluated for the potential reduction of the blue WF. Annual WF from primary crops in the basin amounts to 19,255 MCM/yr (70 % green, 21 % blue and 10 % grey WF, respectively). Banas basin is a net exporter of agriculture commodities with nearly 7391 MCM/yr of water flowing out of the basin due to agricultural exports of which approximately 265 MCM/yr is virtual blue water outflow. Crops having low economic water productivity of blue water are being grown in vast areas resulting in a high blue WF. The optimizing the cropping pattern can result in a 5–42 % lower blue water footprint with 11–39 % higher economic output under different scenarios with and without considering the consumption needs. Changing the cropping pattern and making trade plan to optimize the crop import/exports can be viable option for tackling the blue water scarcity issues in the basin. WF can be managed sustainably by improving water resource allocation for better economic, social, and environmental productivity and going for less aggressive agricultural production.

## Highlights

• We assessed the potential to reduce the blue WF by altering cropping patterns in Banas Basin.
• Annual WF in the BRB over the study period was 19,255 MCM/yr (70 % green, 21 % blue and 10 % grey WF, respectively).
• Banas basin is a net exporter with nearly 7,391 MCM/yr of virtual water ouflows of which 265 MCM/yr is blue water.
• Optimizing the cropping pattern can result in a 5–42 % lower blue WF with 11–39 % higher economic output.

## 1 Introduction

Over the past five decades, global agricultural production has witnessed a remarkable growth, surging by 2.5–3 times over the years [1]. This surge can be attributed in significant part to the expansion of irrigated areas, which have more than doubled in size and have contributed over 40 % to the overall increase in food production. In this context, water has emerged as a paramount requirement for agricultural and food production processes, accounting for a substantial 85 % share of the world's total freshwater consumption [2]. Remarkably, irrigation alone constitutes nearly 70 % of the planet's freshwater usage [3]. This escalating demand for agricultural products to sustain an ever-expanding global population has necessitated the persistent intensification and enlargement of agricultural activities over the preceding decades [4].

The concept of the water footprint (WF) offers a smeans to measure the overall volume of freshwater required for the manufacturing and consumption of goods and services. It is assessed at the point of production and encompasses three key components: green WF (derived from rainwater), blue WF (associated with surface or groundwater), and grey WF (pertaining to water required to assimilate contaminants) [5]. Water security is a pivotal factor driving social and economic progress leading to health improvement, well-being promotion, and economic development [6]. The assessment of WF in agriculture plays a pivotal role in unraveling the impacts and constraints that underlie prevailing crop production systems. Notably, two main facets contribute to virtual water content (VWC): internal WF, gauging freshwater consumption within a specific area to yield goods and services for its inhabitant, and external WF, quantifying imports of freshwater-intense products for consumption from external regions [7,8].

The multifaceted nature of WF is profoundly influenced by the scale, consumption, and production context [9]. WF materializes in varied forms, exhibiting topographical and transient variations across diverse scales, including national, regional, and basin dimensions. Poignantly, agriculture shoulders the lion's share, contributing to a staggering 92 % of humanity's total water footprint [10]. Studies have revealed that 78 % of the worldwide agricultural WF is attributed to green WF, with blue and grey WFs contributing 12 % and 10 %, respectively [11,12]. When comparing WF in rain-fed and irrigated agriculture, with the former accounting for a WF of 5173 BCM/yr (91 % green, 9 % grey) and the latter being 2230 BCM/yr (48 % green, 40 % blue, 12 % grey) highlighting prominence and importance of rainfed agriculture worldwide [13]. In light of these facts, the imperative to establish comprehensive water policies for managing crop WFs across various scales becomes evident, alongside the discernment of optimal water use through the lens of green, blue, and grey WF contributions.

The significance of shifting cropping patterns to address these challenges warrants particular emphasis. Notably, altering cropping patterns presents a plausible avenue for sustaining crop production and mitigating water scarcity, especially given the escalating constraints posed by arid and semi-arid regions, along with the impending specter of climate change [12,[14], [15], [16], [17], [18], [19], [20], [21], [22], [23]]. As the agricultural sector confronts the daunting task of nourishing an expanding global population within the confines of finite natural resources, the adaptive strategy of adjusting cropping patterns emerges as a potential solution. This study presents a comprehensive assessment of VWF and WF of major crops within the Banas River Basin, leveraging locally available datasets. Moreover, this research seeks to optimize cropping patterns by employing a linear programming approach, with the dual objectives of curbing blue water consumption and enhancing the economic efficiency of blue water utilization.

## 2 Materials & methods

### 2.1 Study area

**Fig. 1:**
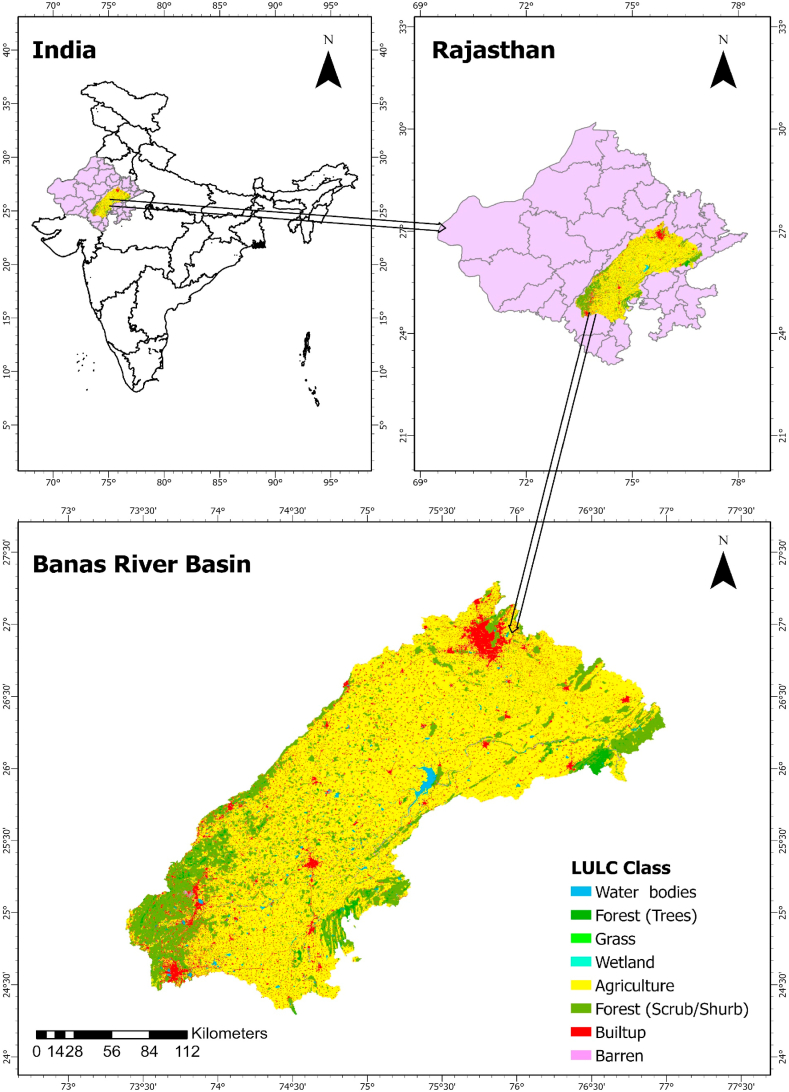
Location of banas river basin.

Banas River Basin (BRB) is located in the eastern part of Rajasthan and occupies a significant area east of the Aravali Mountain range (24°15′-27°20′ lat and 73°25′-77°00′ long) as shown in Fig. 1. BRB has a catchment area of 4.7 Mha within the state of Rajasthan flowing from west to east then merging into Chambal river near Sawai Madhopur [24]. We obtained district-level datasets covering annual statistics on production, productivity, and the cultivated and irrigated areas for various crops from 2008 to 2020, focusing on the districts within the Banas basin from the Agriculture Statistics Handbook published by the Government of Rajasthan. Our analysis concentrated on sixteen major crops grown in the BRB, chosen due to their significant contribution to the total cultivated and irrigated areas, accounting for 94.0 % and 89.6 % annually, respectively.

#### 2.1.1 Water footprint assessment

The estimation of the WF was carried out following WF network guidelines, using the Plug-in version of AquaCrop model 6.0 [25]. Parameterization and calibration guidelines from FAO were followed for simulating various crops [26]. The study employed several datasets, which are detailed in Table 1. In order to account for spatial variations and minimize the number of required simulations, the basin was divided into homogeneous land units (LU) based on factors such as land use, soil, and agro-climatological characteristics [27]. To achieve this, different thematic layers, including soil, AESR, LULC, basin boundaries, and district boundaries, were overlaid, and LU polygons were generated for each district using the intersect feature in ArcGIS [28].

**Table 1:** Various datasets used for determining VWF.

S.No	Type of data	Source
1.	Digital Elevation Model	SRTM DEM, NASA (https://earthexplorer.usgs.gov/).
2.	Agro-Ecological Regions map	NBSSLUP, ICAR (http://geoportal.icar.gov.in/)
3.	Soil properties	Harmonised world soil database v1.2 (http://www.fao.org/)
4.	Land use land cover map	Bhuvan, National Remote Sensing Centre (NRSC), Indian Space Research Organisation (ISRO) (https://bhuvan.nrsc.gov.in/)
5.	District-wise cropped area and agriculture statistics	Agriculture Statistics Handbook, Directorate of Economics & Statistics, Department of Planning, Government of Rajasthan (https://agriculture.rajasthan.gov.in/) and Agriculture Statistics at Glance, Minister of Agriculture & Farmers Welfare, Government of India (https://agricoop.nic.in/)
6.	Per capita consumption of crop and crop products	National Statistical Organisation, National Sample Survey Office (NSSO), Ministry of Statistics and Programme Implementation, Government of India (https://mospi.gov.in/)
7.	Metrological Data	India Meteorological Department (IMD), Ministry of Earth Sciences, Government of India (GOI). (http://www.imdpune.gov.in/) and Modern-Era Retrospective analysis for Research and Applications, Version 2 (MERRA-2), NASA (https://power.larc.nasa.gov/)

**Fig. 2:**
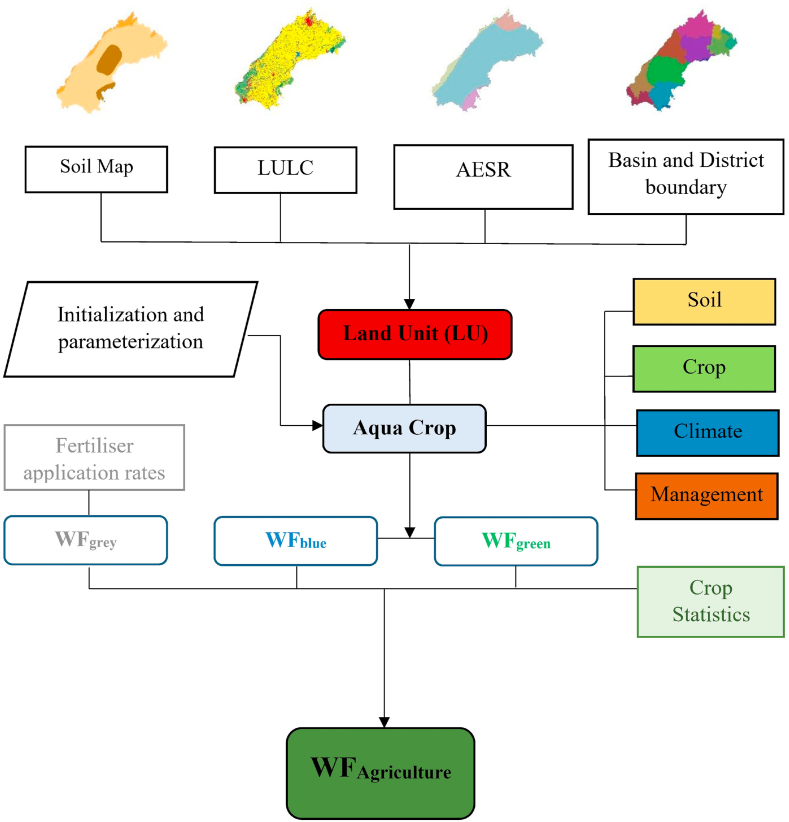
Water footprint assessment methodology (Adopted from Ref. [28]).

Blue and green WFs were determined based on their individual contribution in crop water use (CWU) and dividing the CWU by yield (Y) of crop. Grey WF represents the quantity of freshwater needed to assimilate pollutants, with consideration of natural baseline concentrations and the prevailing ambient water quality criteria. To determine the grey WF the actual fertiliser load from N fertiliser use was multiplied by the leaching-runoff fractions which were determined from local datasets. Methodology of water footprint assessment is illustrated in Fig. 2 and detailed methodology of WF assessment can be referred from Ref. [28].

### 2.2 Virtual water flow

VWF in the basins were calculated as the product of VWC/WF of crop and the quantity of the product exported/imported in the basin. Total water use of crop produce includes consumption requirement of humans, seed required and wastage at farm and market levels. The consumption of agricultural commodities in the basin was determined by multiplying the urban and rural populations with state-level per capita consumption for 30 major crop products obtained from NSSO household estimates [29]. All primary produce is not consumed as such, it needs some further processing which involves losses. Consumption was calculated by dividing the quantity of processed products consumed by their product fraction [30]. Product fraction (pf) is the quantity of the output product (kg) obtained per unit quantity of input product (kg) consumed in the process. Annual virtual water exports were estimated by subtracting total consumption within the basin from the total commodity production in a basin, considering additional quantity as exports. Where consumption of a crop was based on the per capita consumption data on processed crop products and using the following equation (1).

(1) $$Q_{cons.}=\frac{P_{rural}\times q_{rural}+P_{urban}\times q_{urban}}{{pf}_{i}}$$

where,

$$Q_{cons.}:\text{Quantity}\;\text{of}\;\text{crop}/\text{crop}\;\text{product}\;\text{consumed}\left(\text{ton}/\text{year}\right)$$

$$P_{rural}:\text{Rural}\;\text{Population}$$

$$P_{urban}:\text{Urban}\;\text{Population}$$

$$q_{rural}:\text{Rural}\;\text{consumption}\left(\text{ton}\;\text{per}\;\text{capita}\;\text{per}\;\text{year}\right)$$

$$q_{urban}:\text{Urban}\;\text{consumption}\left(\text{ton}\;\text{per}\;\text{capita}\;\text{per}\;\text{year}\right)$$

$${pf}_{i}:\text{Product}\;\text{Fraction}$$

Seed consumption was estimated by multiplying recommended seed rate for different crops with the gross cropped area under the crop. The post-harvest losses were added to consumption, taking 10 % as a fixed percentage of the loss considering wastage at farm and market levels conjunctively [31]. Virtual water balance for agricultural commodities is given as (2,3 and 4):

(2) $${WF}_{cons.}=VWC\times Q_{cons.}$$

(3) $${WF}_{prod.}=VWC\times Q_{prod.}$$

(4) $${VWB=WF}_{cons.}-{WF}_{prod.}$$

where,

$$Q_{cons.}:\text{Quantity}\;\text{of}\;\text{crop}/\text{crop}\;\text{product}\;\text{consumed}\left(\text{ton}/\text{year}\right)$$

$${WF}_{cons.}:\text{WF}\;\text{of}\;\text{crop}/\text{crop}\;\text{product}\;\text{consumed}\left(\text{MCM}/\text{year}\right)$$

$${WF}_{prod.}:\text{WF}\;\text{of}\;\text{crop}/\text{crop}\;\text{product}\;\text{produced}\left(\text{MCM}/\text{year}\right)$$

VWB: Virtual Water Balance (MCM/yr).

#### 2.2.1 Economic water productivity of blue water (EWP_bw_)

EWP_bw_ is a valuable indicator for used ascertain the economics of blue water use in crop production. EWP_bw_ is the ratio of gross returns (₹) to blue WF (m^3^) of a crop production within the basin (5). EWP_bw_ and WFs can help improve the water resources planning and management by identifying the low-value water-intensive crops and help in optimizing future cropping patterns for maximum economic returns and water saving. It is given by,

(5) $${EWP}_{bw}=\frac{Gross\;returnsof\;particular\;crop\left(₹/ton\right)}{Blue\;water\;footprint\left(m^{3}/ton\right)}$$

The average farm harvest price (FHP) of the commodity in Rajasthan state was taken as its market price (Dept. of Agriculture, Gov. of Rajasthan). It is the average wholesale price at which the producer disposes of the commodity to the trader at the market. For crops where FHP was not available Minimum support price (MSP) was used (Agriculture Statistics at Glance, MOA&FW, GOI).

### 2.3 Changing cropping pattern

The cropping pattern pertains to the distribution of cultivated land among various crops during a year. Optimizing the crop planting pattern is crucial for promoting water use sustainability especially in arid and semi-arid areas [32]. Cropping patterns can be an inexpensive and good alternative to current practices which can be adopted at large scale without much difficulty. Linear Programming (LP) was used to optimize the cultivated area under irrigation for the selected crops in the basin using the simplex method available with Open Solver add-in in MS Excel [31]. It was used to maximize the objective function, which was improving the economics of blue water use and reducing blue water consumption in crop production while considering a set of pre-defined constraints of irrigated and total cultivated areas being constant. Different scenarios were evaluated to determine an optimized cropping pattern for the Banas River Basin (Table 2).

**Table 2:** Different strategies evaluated for potential reduction blue WF.

Sr. No	Scenario	Strategy implemented
1.	Current Situation	Current situation in the basin considering the blue water footprint of sixteen major crops.
2.	Scenario I	Minimizing the annual blue water footprint of the basin without considering the consumption needs of the region.
3.	Scenario II	Maximizing total economic value of production without considering the consumption needs of the basin.
4.	Scenario III	Minimizing the annual blue water footprint of the basin while considering the consumption needs of the region.
5.	Scenario IV	Maximizing total economic value of production while considering the consumption needs of the basin.

## 3 Results

### 3.1 Water footprint of crop production

Crop WF (m^3^/ton) under irrigated and rainfed condition was determined using WFN guidelines [5]. Blue, green and grey WF of major crops grown in the basin under different conditions and its comparison with earlier studies is given in Table 3. WF for most crops in the BRB exceeded the global averages which can attributed to lower yield and climatic variation [30]. Individual crop WFs were multiplied with their respective production estimates to determine total WF of crop production. Annual WF of major crops in the BRB over the study period was 19,255 MCM/yr (70 % green, 21 % blue and 10 % grey WF, respectively). Rainfed agriculture contributes to nearly 63 % of total annual WF of crop production of the basin and reason for higher green WF as rainfed agriculture is prominent in the BRB while irrigated agriculture accounts for only 37 %. Blue water contribution in irrigated agriculture is at about 55 %. Wheat and Rapeseed-Mustard crops which are major crop of winter/rabi season accounting for 82 % of irrigated area makes up almost 87 % (67 and 20 %, respectively) of the average annual blue WF. Large WF of crops is directly linked to their crop production and cropped area alongside average WF with crop having higher area under production accounting for high WF in general. These results are in line with results reported earlier for these crops with our results being little higher in most cases due to lower yield and arid climate [[33], [34], [35]].

**Table 3:** WF of major crops grown in the basin and its comparison with earlier studies.

	Irrigated			Rainfed		Current Study Average	Kampman, (2008)	Mekonnen and Hoekstra, (2011)
	WF_blue_ (m³/ton)	WF_green_ (m³/ton)	WF_grey_ (m³/ton)	WF_green_ (m³/ton)	WF_grey_ (m³/ton)			
**Bajra**	1220.7	3185.7	404.2	4511.1	504.0	4912.8	4222	4478
**Barley**	785.1	556.2	176.2	1054.7	212.6	1392.4		1423
**Cotton**	1571.7	1818.9	171.2	2523.4	184.5	3134.9	10,633	4029
**Gram/Chick pea**	1235.3	1700.9	483.7	2180.2	518.4	3059.3	2071	4177
**Groundnut**	2319.9	3458.7	371.8	5681.5	492.3	6162.2	4372	2782
**Guar/Cluster beans**	1494.8	4568.8	579.7	5497.9	614.3	6377.8		
**Jowar/Sorghum**	2118.3	5102.4	559.7	5834.1	581.6	7098	3589	3048
**Lentil/Masoor**	2881.1	2225.1	528.6	3898.8	553.6	5043.7		5874
**Maize**	1170.3	3184.0	313.5	3700.6	326.9	4347.6	2399	1222
**Moong/Mungbean**	2268.9	7610.1	1061.6	8481.2	1084.4	10253.1		
**Rapeseed & Mustard**	1226.2	1569.2	440.1	2041.6	465.7	2871.4	3972	2809
**Rice/Paddy**	2095.1	2481.0	270.5	5318.8	404.8	5285.1	4073	1673
**Sesame**	3200.1	11539.5	1331.3	12809.0	1384.3	15132.1		9371
**Soybean**	2146.5	4021.8	400.6	5230.3	423.8	6111.5	3526	2145
**Urad/Black Gram**	2807.9	7960.7	1011.3	9221.7	1044.4	11023.0		
**Wheat**	1264.1	446.1	136.7	1380.3	155.8	1691.5	1412	1828

**Fig. 3:**
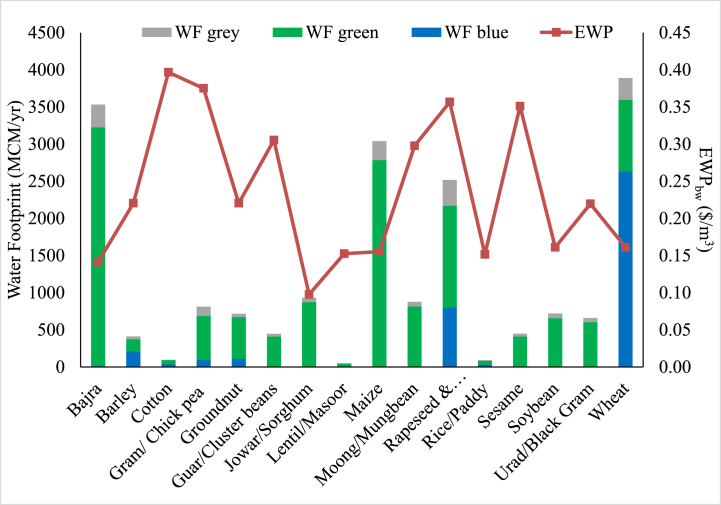
WF of crop production and EWP_bw_ of major crops in BRB.

WF and EWP_bw_ for major crops in BRB is presented in Fig. 3. It was found that a considerable portion of accessible blue water is being used to produce low value-water intensive crops. Cotton, Gram/Chickpea, Rapeseed-Mustard and Sesame were among the crops having a higher value EWP of blue water (0.4, 0.38, 0.36 and 0.35 $/m^3^, respectively taking 1$ = 74 ₹). Jowar/Sorghum, Bajra/Pearl millet, Rice/Paddy and Lentil/Masoor were among the crops having a lower EWP of blue water (0.1, 0.14, 0.15 and 0.15 $/m^3^, respectively). Among the crops with lower EWP, some crops are already mainly grown under rainfed conditions.

Among the crops with a high blue WF of crop production namely Wheat (2630 MCM/yr), Rapeseed-Mustard (800 MCM/yr), Barley (210 MCM/yr), Groundnut (112 MCM/yr) and Gram/Chickpea (96 MCM/yr). Wheat, Barley and Groundnut have a lower blue water EWP (0.16, 0.22 and 0.22 $/m^3^, respectively). While, Rapeseed-Mustard and Gram/Chickpea have higher EWP of 0.36 and 0.38 $/m^3^, respectively.

Despite having low EWP, the crops like Wheat, Barley and Groundnut are being grown in the BRB in vast areas, mainly because these are either staple food crops or main cash crops of the basin having lower production cost/labour requirement. Low value water-intensive crops like chickpea, mustard and pearl millet are grown in considerable part of Gomati River basin was major reason behind its high blue WF [36]. It was suggested that confining these crops to rainfed conditions as they can have same yield even under rainfed conditions may result in considerable blue water savings.

Groundwater is pumped freely in the basin as per the demand for irrigating crops due to subsidized electricity and inefficient irrigation management practices. In the absence of any effective policy measures, the basin will face severe water scarcity and environmental degradation in the future. Based on the long-term groundwater levels trends in the BRB it is expected that 63 % of the observation wells will become dry by the year 2040 during the pre-monsoon season and 43 % of the observation wells will be dry during the post-monsoon period in the future [24]. EWP and WFs can be helpful in improving the water resources planning and management by identifying the low-value water-intensive crops and helping optimize the future cropping patterns for maximum economic returns and water saving.

### 3.2 Virtual water flows

VWF in the BRB averaged over the study period are given in Table 4. Banas basin is a net exporter of agriculture commodities. On average, nearly 7391 MCM of virtual water flows out of the Banas basin due to agricultural exports annually. Similarly, there is 265 MCM of virtual blue water outflow annually from exports. Rapeseed-Mustard makes up a large part of the annual virtual blue water flow (582 MCM/yr), followed by Barley (201 MCM/yr). Maize, Rapeseed and Mustard, and Bajra make up a large portion of exports from the basin, accounting for nearly 83 % of annual virtual water outflows.

**Table 4:** Average Annual Virtual water flows in Banas River Basin.

Crops	Production	Consumption	Crop Export/Import	Virtual Water Flow	Virtual Blue Water Flow
	ton	ton	ton	MCM/yr	MCM/yr
**Bajra/Pearl millet**	719,590	332,405	−387,185	−1900	−6.3
**Barley**	284,449	11,199	−273,250	−395	−200.9
**Cotton**	29,026	493,729	+464,703	+1556	+588.7
**Gram/Chickpea**	286,234	65,332	−220,901	−749	−72.3
**Groundnut**	115,243	60,183	−55060	−339	−49.6
**Guar/Cluster beans**	72,806	12,902	−59904	−368	0.0
**Jowar/Sorghum**	153,911	7942	−145,968	−883	−0.1
**Lentil/Masoor**	11,327	44,333	+33,006	+149	0.0
**Maize**	780,004	165,046	−614,959	−2397	−5.7
**Moong/Mungbean**	96,107	32,143	−63964	−584	0.0
**Rapeseed and Mustard**	808,455	215,528	−592,927	−1843	−582.2
**Rice/Paddy**	18,168	110,341	+92,173	+466	+157.8
**Sesame**	31,485	59,738	+28,253	+404	+0.1
**Soybean**	128,410	164,226	+35,817	+201	+1.5
**Urad/Black Gram**	66,898	9424	−57474	−567	0.0
**Wheat**	2142318	2063944	−78374	−142	−96.1
**Virtual water balance**				−7390.8	−265.2

^a^+ sign indicates import of virtual water and “-” sign indicates export.

**Fig. 4:**
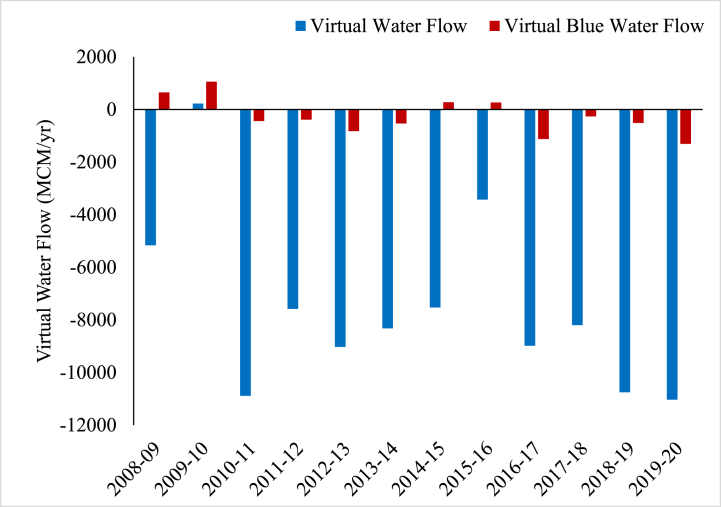
Virtual water flows in BRB.

VWF and its blue component for major crops in the BRB over the years is presented in Fig. 4. In a similar study conducted in India, Gomti river basin was found as a net importer of virtual water while Betwa basin was termed as an net exporter of virtual water [17,34,36]. Virtual water outflows from BRB advocates the need for local level planning and management of water resources at the river basin scale. Optimizing the allocated area under different water-intensive crops without compromising the consumption needs within the basin can be suitable option to tackle water scarcity issues by cutting the surplus production [36]. Low value water-intensive crops are being grown in a considerable area in the BRB resulting in high WF and despite being a water scare region significant amount water is being exported out of basin. From observing the current situation in BRB we can see a clear need for developing water allocation policies and adopting crop planning in the region.

### 3.3 Optimizing cropping pattern

Under the prevailing cropping pattern, a substantial quantity of freshwater is being utilized in the cultivation of crops with low EWP_bw_. Efficient use of blue water resources can be achieved by allocating water resources to various crops based on the EWP_bw_. Finding the optimal cropping pattern needs sophisticated optimization tools and comprehensive assessments of production and consumption demands as well as water use. Linear Programming was used to optimize the cultivated area under irrigation for the selected crops in the basin. The solution to the linear programming model was obtained using the Simplex method available with the Open Solver add-in in MS Excel. The objective function was to maximize the economics of blue water use or minimize the blue water consumption in crop production in irrigated areas as per the scenario. Constraints were set as the total and irrigated area will remain constant. For scenarios III and IV, the optimal cropping patterns suggested were obtained by giving the blue water reduction importance without compromising basin consumption needs. Optimized cropping patterns were developed based on the data for the year 2019–2020. Different scenarios were evaluated to see how changing the cropping pattern could potentially reduce the blue WF of the basin.

**Fig. 5:**
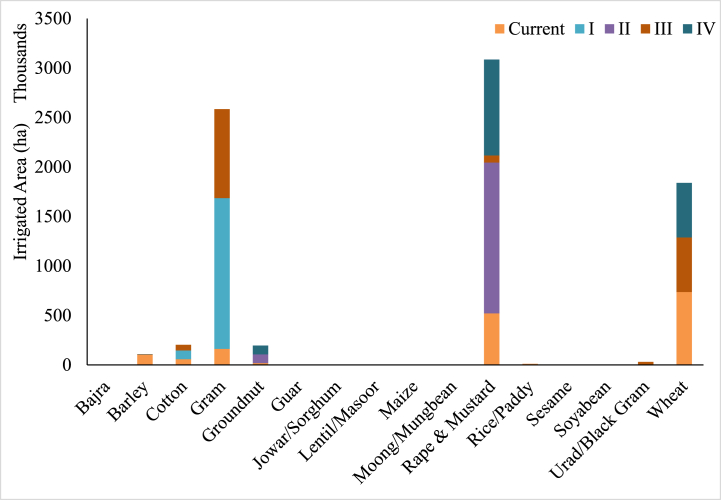
Irrigated area of various crops under different scenarios.

Irrigated area under the current and optimized cropping patterns for major crops under different scenarios is presented in Fig. 5. Under the optimized cropping patterns, the irrigated areas under Bajra, Barley, Jowar and Wheat crop was reduced as these crops had lower EWP_bw_. For scenario I, there was significant reallocation of irrigated area from other crops to Gram followed by cotton as it had the lowest blue WF. In scenario II, focus was on Groundnut and Rapeseed-Mustard crop as they had high EWP_bw_. Under scenario III, consumption of the basin was considered hence irrigated area was distributed among the Gram, Wheat and Cotton to optimize the blue WF. For scenario IV, main focus was on maximizing the economic value of production without compromising the consumption needs of the basin emphasis was on allocating the irrigated area to Wheat and Rapeseed-Mustard crop as Wheat is a staple crop with high consumption and Rapeseed-Mustard had high EWP_bw_. Although the suggested cropping patterns are promising in terms of reducing the blue WFs and maximizing the economic value of production, this needs to be seen in view of various social and technical aspects. When developing management plans, it is essential to consider factors such as the availability of capital and labor. Current agricultural policies grant farmers the autonomy to cultivate crops according to their preferences. Consequently, it is harder to convince farmers' to shift towards other crops as reallocation of crops entails various technical and economic considerations.

**Fig. 6:**
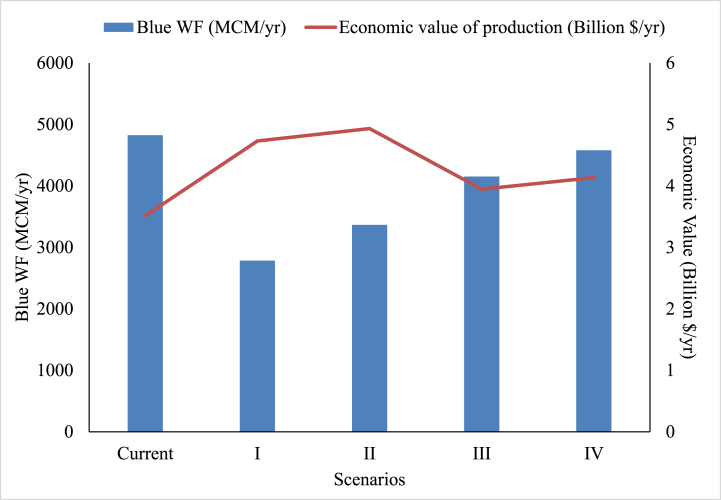
Blue WF and economic value of production under different scenarios. (For interpretation of the references to colour in this figure legend, the reader is referred to the Web version of this article.)

Blue WF and economic value of production under different scenarios is presented in Fig. 6. In comparison to current scenario, scenario I resulted in 42 % lower blue WF and 33 % higher economic value of production in the basin. This scenario was most promising in terms of reducing blue WF, but this comes with compromising the food security of the region. In scenario II, there was 30 % blue water saving with 39 % higher economic value of production. This cropping pattern gives the maximum economic value of production at $ 4.9 billion/₹ 36,418 crores (Taking 1$ = 74 ₹) by giving preference to crops with high EWP_bw_. But this too comes at a compromise of the food security of the region. When we considered the consumption needs of the basin and optimized cropping pattern to minimize the blue WF (Scenario III) we found 13.8 % blue water saving with 11 % higher economic value of production in comparison with current situation. Similarly, under scenario IV we observed a 5 % reduction in blue WF with a 16 % increase in the economic value of production without compromising the consumption needs. While the blue water saving of 5 % could be considered menial to undertake such arduous task but it should be observed that optimized cropping pattern under current study is the one obtained under different constraints and not only reduce the blue WF but also increase economic value of production. If restrictions on fulfilling consumption needs of the basin are lifted blue water savings can be significantly increased. In order to achieve this, such water intensive crops and crop products can be arranged from other regions of India or world, but this can hamper self-sufficiency and food security in the basin to some extent. Under the current study focus is on major crops already prevalent in the region which can easily be adopted by farmers with little effort and motivation. Instead of focusing on the prevalent crops we can also introduce new high value less water intensive crops for cultivation in the basin.

## 4 Discussion

By definition, there are two major ways of lowering WF that is by increasing the production in terms of yield which means producing more from same amount of water and second is lower losses by improving irrigation efficiency (*e.g.*, using drip irrigation systems and reducing soil evaporation by applying mulches). Water use and in turn WF can be reduced by reducing non-beneficial consumptive water use by adopting different irrigation management strategies, methods and technologies [37]. Allocating crops from high blue WF areas to low blue WF areas can also result in significant savings in water [38]. There are several other ways to reduce WF including changing planting dates and cropping pattern resulting in lowering fertilizer loss, and more effective use of precipitation [21,[39], [40], [41], [42], [43]]. Adopting in-situ rainwater harvesting and soil moisture conservation measures, while prioritizing crops that make efficient use of green water, can result in significant reductions in blue water consumption [36].

The cropping pattern is a promising option for reducing blue WF in the basin. It can have a significant impact on overall WF for crop production. Changing the cropping pattern can play significant role in reducing water scarcity while increasing food and cash crop production in a basin [44,45]. Basin-level water allocation strategies and optimal cropping patterns can significantly reduce the blue WF in the Gomti and Betwa basins irrigated areas [31]. Implementing drastic actions such as decreasing the cultivation of water-intensive rice in or reducing wheat production in water-scarce regions led to a notable reduction in WF [20]. Optimizing the cropping pattern to reduce water scarcity has significant trade-offs and can hamper food security if not properly planned.

There is need for comprehensive studies to evaluate and address the water scarcity issues by proper policy framework at basin scale. Optimized cropping pattern should be made while considering the consumption needs and demands of the region. Long-term investigation should be undertaken on transitioning from water intensive crops to crops with high EWP_bw_ and lower crop WF. Exports and imports of should be managed in such a way that exports of water intensive crops should be reduced instead they should be imported from other water rich areas. For this there is need for National and Global scale plaining to optimize water use among various regions. Further, research is needed to assess the impact of water-efficient irrigation techniques such as sprinkler or drip irrigation, the application of different fertilizers, and cropping pattern on the WF and EWP so that water used can be reduced without threatening the socio-economic conditions of the region.

## 5 Conclusions

Modelling water footprints help us in identifying the impacts of various strategies on the current crop production systems, evaluating constraints, and aiding us in devising appropriate strategies to enhance water efficiency. Annual WF during the study period for primary crops in the basin amounted to 19,255 MCM/yr, with 70 % being green, 21 % blue, and 10 % grey WF, respectively. Based on virtual water balance Banas River Basin is a net exporter of agriculture commodities. On average, nearly 7391 MCM/yr of water flows out of the Banas basin due to agricultural exports. Of this, approximately 265 MCM/yr is virtual blue water outflow. Rapeseed-Mustard makes up a large part of the annual virtual blue water flow (582 MCM/yr), followed by Barley (201 MCM/yr). Considerable amount of precious blue water resources is being used to produce crops having low economic water productivity. Changing the cropping pattern can be viable option for tackling the blue water scarcity issues in the basin. Scenario I came out a promising option in terms of reducing the blue WF with 42 % lower blue WF and 33 % higher economic value of production in comparison to current situation, but this comes with compromising the food security of the region. Economic value of production is maximized under scenario II, with 39 % higher economic value with 30 % blue water saving but with compromise of consumption needs. If we considered the consumption needs of the basin, 14 % blue water saving with 11 % higher economic value of production was found in scenario III and 5 % reduction in blue WF with 16 % increase in the economic value of production under scenario IV. Changing crop allocation and introducing new high value low water intensive crops can make blue water footprint of the basin more sustainable. This study advocates the need of a trade plan, to optimize the crop import/exports based on the situation of the region instead of exporting water-intensive crops. There is need for a comprehensive irrigation policy to properly manage available water resources and gradually shift the irrigation practices into more efficient ones which are socially acceptable and financially viable.

## Data availability statement

Has data associated with your study been deposited into a publicly available repository?- **No**.

Data will be made available on request.

## CRediT authorship contribution statement

**Mukesh Kumar Mehla:** Conceptualization, Data curation, Formal analysis, Investigation, Methodology, Resources, Visualization, Writing – original draft, Writing – review & editing. **Mahesh Kothari:** Conceptualization, Investigation, Methodology, Resources, Supervision, Writing – review & editing. **P.K. Singh:** Conceptualization, Investigation, Methodology, Resources, Supervision, Writing – review & editing. **S.R. Bhakar:** Conceptualization, Formal analysis, Investigation, Methodology, Supervision, Writing – review & editing. **K.K. Yadav:** Formal analysis, Investigation, Methodology, Supervision, Writing – review & editing.

## Declaration of generative AI and AI-assisted technologies in the writing process

During the preparation of this work the author(s) used AI tools for language editing and improving articles readability. After using this tool/service, the author(s) reviewed and edited the content as needed and take(s) full responsibility for the content of the publication.

## Declaration of competing interest

The authors declare that they have no known competing financial interests or personal relationships that could have appeared to influence the work reported in this paper.
